# Factors associated with foreign-educated nurses’ willingness to continue working in Japan: a qualitative study

**DOI:** 10.1186/s12912-024-01890-4

**Published:** 2024-04-03

**Authors:** Kazuko Tanaka, Koichi Yoshimura

**Affiliations:** 1 Faculty of Nursing and Human Nutrition, Department of Nursing, Yamaguchi Prefectural University, 6-2-1, 753-0021 Sakurabatake, Yamaguchi Japan; 2 Graduate School of Health and Welfare, Yamaguchi Prefectural University, 6-2-1, 753-0021 Sakurabatake, Yamaguchi Japan

**Keywords:** Diversity, Economic partnership agreement, Foreign-educated nurses, Japan, Willingness to work

## Abstract

**Background:**

Japan has been accepting foreign nurse candidates since 2008 under Economic Partnership Agreements (EPAs). As globalisation progresses, nurses from diverse backgrounds are expected to play an active role in the medical field. Using an interview survey, this study examined the factors associated with EPA nurses’ willingness to continue working in Japan.

**Methods:**

We conducted semi-structured interviews from January 2022 to July 2023 with eight EPA nurses and one EPA nurse candidate working in Japan to investigate the factors associated with foreign-educated nurses’ willingness to continue working in Japan. The interview guide included items on the status of the daily performance of their duties, what they found pleasurable in their nursing experience in Japan, difficulties they encountered in carrying out their nursing duties, and their expectations of the Japanese staff around them. Data were analysed using thematic analysis.

**Results:**

From the interview data, seven themes were extracted. To continue working in Japan, it was important for EPA nurses to be able to communicate with patients and colleagues, maintain self-esteem and motivation, be resilient, have support from EPA peers and family members, be accepted by others such as patients and colleagues, and be satisfied with the support they received.

**Conclusion:**

The EPA nurses experienced many difficulties after becoming nurses and tended to be isolated because of their non-Japanese status. The results suggest that not only support from colleagues and supervisors but also a general understanding of EPA nurses from Japanese society is necessary. As globalisation accelerates, the Japanese nursing field needs to understand the diversity of the nursing profession and build a support system that will enable them to continue to take pride and feel motivated in their work.

**Supplementary Information:**

The online version contains supplementary material available at 10.1186/s12912-024-01890-4.

## Background

Japan has accepted nursing candidates from three countries under Economic Partnership Agreements (EPAs). In the ‘New Growth Strategy’ approved by the Cabinet in 2010, Japan is committed to increasing the number of foreign nationals working in hospitals and long-term care facilities by ensuring the acceptance of nurse and care worker candidates based on EPA, and promoting the development of medical and long-term care skills and knowledge in Japan [1]. In Japan, the number of foreign residents reached an all-time high in 2022, and the medical needs of foreigners are expected to increase, making it urgent to secure diverse human resources in the nursing field. The percentage of foreign residents by nationality was 15.9% for Vietnam, 9.7% for the Philippines, and 3.2% for Indonesia, where the EPA nurses are from, with the three countries accounting for nearly 30% of the total [2]. EPA nurse candidates are accepted from these three countries. The increase in the number of foreign residents poses a major challenge in providing nursing care to patients with different medical beliefs and practices [3], and recruiting EPA nurses may be beneficial in addressing these issues.

However, Japan has a short history of accepting foreign-educated nurses, and there is little research on foreign nurses. Research on EPA nurses has focused on policy issues related to the Japan-Philippines EPAs on nurse migration and the perspective of hospital administrators in the host country, Japan. Yagi pointed to the failure of the Japan-Philippines EPA’s health worker immigration provisions due to the low passing rate of Filipino nurses in the national nursing exam and advocated adopting a USA-based approach to licensing examinations [4]. Hirano reported the factors associated with the recruitment of EPA nurses among managers of hospitals in Japan who have not previously employed these nurses. According to the study, more than 90% of hospital managers felt that training EPA nurses would be difficult. Half of the hospital managers were considerably interested in Japan’s policy on recruiting EPA nurses, although only 20% intended to recruit EPA nurses in the future [5]. A systematic review of studies on foreign-educated nurses in Japan found that EPA nurses and nurse candidates face many challenges and difficulties, including language and communication barriers, low passing rates in national nursing exam, adaptation to the workplace, unfamiliar social environment, and psychological distress. The review highlights the urgent need for policymakers to take immediate action in addressing these challenges [6].

A qualitative study also identified Indonesian nurses’ experiences living in Japan. In a study of Indonesian nurses’ experiences of living in Japan, Indonesian nurses reported that they experienced communication difficulties, differences in nursing practice and culture from their home country, and that the national nursing exam as the culmination of their studies was difficult. On the other hand, they reported wanting a better life than before and enjoyed living in a developed country [7]. However, there are few qualitative studies from the perspective of Vietnamese and Filipino EPA nurses. Furthermore, no research has focused on foreign-educated nurses’ willingness to continue working in Japan.

In recent years, there has been an increase in the passing rate of EPA nurse candidates in the national nursing exam, which reached 22.4% in 2023. However, this percentage is still significantly lower compared to the national passing rate of 90.8% [8]. Even after passing the national exam, it is reportedly difficult for foreign nurses to perform their duties in Japan without facing problems because of their lack of Japanese language skills and nursing knowledge [9]. Indeed, EPA nurses returning to their home countries after a few years, even after passing the national exam, has become a significant problem [10]. In principle, EPA candidates who enter Japan under an EPA must terminate their employment contract and return to their home country when their three-year (nurse candidates) stay expires if they do not pass the national exam. However, a system is now in place for those who return to Japan after failing the national nursing exam to retake the exam [11].

The importance of diversity among nurses and healthcare professionals has been reported outside Japan. Nurses comprise the largest percentage of human resources in the healthcare field and are considered to be in a critical position to meet the diverse needs of patients and contribute to reducing health disparities. Therefore, nurses need to take the lead in bridging the diversity gap in the healthcare workforce, and there is a need to increase the diversity of students and faculty in the future [12].

Increasing diversity in nursing has also been suggested as an important strategy to foster equality in healthcare access and delivery [13]. However, working in a culturally diverse team with patients from different cultural backgrounds can be challenging due to differing opinions, beliefs, thoughts, norms, customs, and traditions [14]. Poor management of cultural diversity has been reported to lead to maladaptive behaviours [15] and interpersonal conflicts [16]. Wesołowska reported that cross-cultural empathy may protect against perceived time pressure at work, distress, and sleep problems among native and foreign-born nurses in culturally diverse healthcare settings [14]. Another study showed that foreign-educated nurses value the recognition and appreciation of patients, colleagues, and employers [17].

There is a global shortage of nurses, and in developed countries, the number of foreign-educated healthcare professionals has increased by 60% since 2010 [18]. Nursing is based on treating people as individuals, recognising diversity, and respecting dignity and human rights [19], which also applies to the nursing workforce. Few studies have clarified the factors that cause foreign nurses to continue working in Japan and the support they seek. By clarifying these factors, we can obtain suggestions for supporting foreign nurses; this perspective has inspired the current study. Furthermore, there is a possibility that such an investigation could contribute to ideas and recommendations for the retention of foreign nurses in Japan.

In Japan, there is a growing momentum to accept new foreign workers to cope with the labour shortage caused by the declining birthrate and ageing population. Accepting foreign nurses can be the first step in inclusion efforts to create opportunities and a climate of mutual recognition and acceptance of diverse human resources.

This study aimed to explore the factors associated with EPA nurses’ willingness to continue working in Japan through an interview survey.

## Methods

### Design

This study used a qualitative descriptive research design to explore the factors associated with EPA nurses’ willingness to continue working in Japan. The reporting of the qualitative results adhered to the COREQ guidelines.

Qualitative research is based on interpretation, which requires inputs from researchers. Although the interpretation is subjective, the authors of this study are well-qualified to understand the nuances of the topic. The first author (KT) is a female Japanese nursing associate professor at a university with nursing and midwifery experience in Japan and a foreign country. KY is a Doctor of Medicine with extensive domestic and international healthcare human resources knowledge, a joint research promotion program director, and a university vice president.

### Setting

The study site is a regional city located in Western Honshu, Japan. The number of foreign residents has slightly decreased in recent years owing to entry restrictions imposed because of the COVID-19 pandemic; except in this scenario, the number of foreign residents in this city has generally been rising. In particular, the number of people from Vietnam and the Philippines has increased. According to the statistical data for the subject area in 2023, Vietnamese accounted for 23.7% of the total, second only to Koreans at 24.2%. Filipinos accounted for 10.0%, the fourth largest share after Chinese at 12.6%. Compared to the number of foreign residents in 2013, the number of Vietnamese residents has increased by about 12 times, while the number of Filipinos has doubled, indicating that the number of Vietnamese and Filipinos has increased rapidly over the past decade [20]. Since the start of the EPA nurse acceptance programme, a total of 1644 nurse candidates have arrived in Japan by FY2022. However, in January 2023, only 304 are currently employed in Japan, 22 of whom work in the area studied [21]. The area has several facilities that accept EPA nurses. In addition, the area is promoting various efforts to develop an inclusive community wherein both Japanese and foreigners can be active members. However, it is also a depopulated area with a declining population.

### Sample recruitment

The participants were foreign nurses under EPA working at medical facilities in Japan. A written request for research cooperation was sent to the person in charge of the list of names of facilities where EPA nurses who had passed the national nursing exam were working, which was published on the Ministry of Health, Labour and Welfare’s website [22, 23]. The director of nursing of the approved facilities introduced us to the EPA nurses or EPA nurse candidates. The data were collected from January 2022 to July 2023.

### Data collection

Semi-structured interviews were conducted with eight EPA nurses and one EPA nurse candidate working at four facilities to investigate the factors affecting EPA nurses’ willingness to continue working in Japan. We developed an interview guide for this study with reference to previous research on EPA nurses. The researchers (KT, KY) designed the first draft of the interview guide and then discussed with members of the joint research promotion program (Supplementary material 1). The interview guide was tested on the women with overseas nursing experience. Prior to the interview survey, we collected information from the participants about their background, including age, family members living with them, religion, most recent education, length of stay in Japan, nationality, Japanese language proficiency level, years of nursing experience in their home country and in Japan, time until passing the national nursing exam, and the number of times they had taken the exam. The interview guide included items on the status of the daily performance of their duties, what they found pleasurable in their nursing experience in Japan, any confusion or difficulties they encountered in carrying out their nursing duties, and their expectations of the Japanese nurses and supervisors around them when working in Japan in the future. The first author (KT) conducted the semi-structured face-to-face interviews in easy Japanese. The interviews were conducted in a conference room or another location where privacy could be ensured. Two interviews were conducted online because of measures taken against new coronavirus infections. The interview was recorded using a voice recorder, with the participants’ consent, and each interview lasted approximately one hour. Data collection was terminated upon achieving theoretical saturation; no further information emerged after nine interviews.

### Data analysis

The data analysis process was inductive, following Braun and Clarke’s thematic analysis procedure [24]. This analysis comprises six phases: familiarisation, generating codes, generating, reviewing themes, defining and naming themes, and creating the report [24].

After the interviews were completed, we reviewed the responses shared by the participants and checked with them to ensure there were no errors in the researcher’s interpretation. KT and KY repeatedly read the verbatim records to familiarise themselves with the participants’ expressions. Subsequently, two authors (KT and KY) independently coded the data relevant to the current study. Disagreements were discussed with KT, KY. The next step was to group related codes into potential themes (KT, KY). Ongoing inductive analysis was conducted to check whether the themes worked in relation to the coded extracts and the dataset and to refine the overall story. Themes were refined, reviewed and reconciled, repeatedly back to the data to ensure consistency. Triangulation was used to reduce the effect of research bias to establish confirmability. ATLAS.ti software was used to facilitate data analysis.

### Ethical considerations

The researchers adhered to the recommendations of the Declaration of Helsinki in conducting this research. Ethical approval was obtained from Yamaguchi Prefectural University (#2021-22). Before data collection, the purpose of the research was described to the participants. Further, the participants were assured of confidentiality, and all the collected data, including audio recordings and transcripts, were securely stored and accessible only to the research team. Before the interviews, the interviewer explained the study’s aims and the participants’ rights. Written and verbal information was provided to the participants, and written consent was obtained before the interviews. Informed consent was obtained from all participants. Interviews were conducted in a private room where privacy could be ensured, and the dates and times were set according to the participants’ preferences. Interviews were conducted in Japanese, in a slow-paced manner and using simple and clear language. If participants had difficulty answering in Japanese, we explained that they could also respond in English. The interviewers were careful about not extending the interview time excessively and paying attention to the participants’ reactions.

## Results

### Participant characteristics

All nine participants were female, with an average age of 32.2 ± 4.1 years. Six were unmarried, and three were married. All participants worked in medium-sized hospitals with bed numbers ranging from 150 to 400 beds. The hospital provided either rented apartments or dormitories exclusively for EPA staff to stay. All participants received learning support for Japanese language and nursing studies while working as EPA nurse candidates until they passed the national nursing exam and four continued to receive study support from the hospital after passing the national nursing exams. Learning support was provided by Japanese language teachers and hospital nursing staff. The Director of Nursing directly coordinated learning and daily life support. Seven lived alone, one with her husband and one with her husband and children. Five were from the Philippines, and four were from Vietnam. Four participants had N2 Japanese language proficiency, four had N3 Japanese language proficiency, and one did not respond to this item. All the participants had university degrees, including one on a leave of absence from graduate school. The average duration of residence in Japan was 4.4 ± 3.7 years, the average years of nursing experience in their home country was 3.7 ± 1.2 years, and one participant had not yet passed the national nursing exam. The average years of nursing experience in Japan for the eight participants who had passed was 2.8 ± 3.3 years, the average time to pass the national exam was 2.5 ± 1.3 years, and the average number of times the national exam was taken was 2.5 ± 1.1. All the participants were full-time employees (Table 1).

**Table 1:** Characteristics of the Participants

Variable		*N* = 9(%)
Age: mean(SD)		32.2(± 4.1)
Length of stay in Japan: mean(SD)		4.4(± 3.7)
Years of nursing experience in home country: mean(SD)		3.7(± 1.2)
Religion	Catholic	5(55.6)
	Non-religious	4(44.4)
Sex	Female	9(100)
Marital status	Single	6(66.7)
	Married	3(33.3)
Family structure	Living alone	7(77.8)
	Living with husband	1(11.1)
	Living with husband and children	1(11.1)
Educational background	Tertiary education	9(101)
Nationality	Philippines	5(55.6)
	Vietnam	4(44.4)
Japanese language proficiency	N2	4(44.4)
	N3	4(44.4)
	No answer	1(11.1)
Place of work	Ward	9(101)
Type of work	Full-time	9(100)
*Years of experience as a nurse in Japan*: mean (SD)		2.8(±3.3)
* Time taken to pass the national exam *: mean (SD)		2.5(±1.3)
*Number of national exam taken*: mean (SD)		2.5 (± 1.1)

* The average of the eight EPA nurses who have passed the national nursing exam

### Key themes

This qualitative study used an interview survey design to identify seven themes: ‘communication’, ‘self-esteem’, ‘motivation’, ‘resilience’, ‘support from EPA peers and family’, ‘acceptance by others’, and ‘satisfaction with support received’.

Initially, the EPA nurses found communicating with their patients and colleagues difficult. However, they tried to overcome this difficulty through continuous efforts, and they became happy to ‘communicate’ with patients and colleagues, which also improved their self-esteem. These women were proud to have passed the Japanese national nursing exam; however, their self-esteem was sometimes lowered by the strict guidance they received after coming to Japan. It was essential for the EPA nurses to have support from their EPA peers and family members in their own language at times of loneliness and low motivation due to challenging work situations.

EPA nurses were resilient in accepting unfamiliar nursing tasks that differed from those in their home countries, taking on these tasks flexibly. Being accepted by others was important for EPA nurses, and they sincerely appreciated their Japanese supervisors and colleagues who accepted and supported them as they would their patients and family members. Meanwhile, they realised the difficulty of building relationships with Japanese people. In addition, since these women had chosen their respective facilities by prioritising the quality and types of support received, such as whether there was continuous Japanese language study support, they were grateful that there were work-related considerations to ensure sufficient study time. However, dissatisfaction with incentives and support, such as salary and lack of support in their daily lives, led to decreased ‘motivation’. Many of the EPA nurses had come to Japan highly motivated to learn about Japan’s distinguished medical care and nursing services. However, for those who came to Japan without this kind of motivation, their desire to continue working in Japan was uncertain. In addition, in most cases, the EPA nurses felt a strong sense of alienation because there were no foreign-educated nurses among their colleagues in the workplace, and they were the only foreign-educated nurses, which led to a decrease in motivation among the EPA nurses.

### Communication

The EPA nurses had passed the national Japanese language exam, had high language skills, and could communicate effectively; however, they faced difficulties with the technical terms used in their daily work. In addition, communication with patients and their families who spoke dialects was problematic.

Further, many faced difficulties with nursing records and during conferences, even though they could engage in daily conversations. EPA nurses from the Philippines felt that it was easier to work in a country where English was spoken for nursing planning (as is the case in their home country), and the fact that few Japanese nurses could speak English was an additional factor in their sense of difficulty in communicating with their colleagues.

“*I am not used to Japanese, and the nursing lingo is difficult, so I do not understand when I work the night shift or when I work. I do not know everyone’s opinion, and I cannot express my own opinion, so when there is a sudden change in a patient, I am like, ‘What should I do?’ I had no idea what to do. So, I could not do my job, and that was hard for me. I think that is the hardest thing.*” (EPA8)

However, the EPA nurses came to Japan after studying Japanese, and they made efforts to study Japanese continuously and intentionally practised using the language. They were pleased to see that their Japanese language skills were developing, as they could communicate with patients who spoke dialects with whom they had difficulty communicating previously and could express their opinions to their colleagues.

“*At first, when I talked with patients, I sometimes had a hard time understanding their various expressions because of their dialects. I do not understand. “What” I am sorry, I do not understand. At that time, I asked another staff member. That was three years ago, I did not understand at all. But now I understand.*” (EPA5)

### Self-esteem

The EPA nurses were proud that they had been selected from among many applicants to come to Japan to become EPA nurse candidates and that they had passed the Japanese national nursing exam early, within three years of coming to Japan. They were also proud of the clinical nursing experience they had gained so far in their home country.

However, they were subject to some harsh attitudes and verbal guidance in the departments where they were assigned. They understood that they had to perform the same duties as Japanese nurses but strongly desired to be nurtured over time with the understanding that they were foreigners. In some cases, their self-esteem was hurt because they could not perform tasks that they could do without problems in their home country, even though the methods were different from those used in Japan.

“*My friend, who is also working in Japan now, confided in me that she passed the national nursing exam with me this year and has been working as a nurse since then, but her seniors are a bit strict and get angry at her no matter what she does. We are foreigners working in a place where the language is not our mother tongue, so it is natural that we do not understand the language. So, I just need seniors to explain it to us in a gentler way.*” (EPA8)

There were also cases in which self-esteem was damaged because some nurses passed the national exam while others did not, even though they came to Japan simultaneously. In some cases, when only one EPA candidate working at a hospital with other candidates passed the national nursing exam, she was isolated from other nurses who had not passed the exam. There was a clear distinction between those who passed and those who did not, and both had painful experiences.

### Resilience

The duties of EPA nurses in their home countries differ from those of nurses in Japan. In their home countries, it is common for patient’s family members to perform tasks such as assisting with toileting and cleanliness. However, the EPA nurses did not feel uncomfortable with labour-intensive nursing tasks such as assisting with bathing and other heavy lifting that they had not performed in their home countries; they took the initiative and made efforts to carry out tasks involving heavy lifting, gradually becoming accustomed to them. They had a flexible attitude towards accepting Japanese nursing practices for the benefit of their patients and gradually became more independent in tasks with which they were unfamiliar. They felt that there were no unacceptable aspects of the nursing techniques.

“*Of course, it is not only about me but also about Japanese people and their way of thinking. I am from a different country, so it is better to understand their way of thinking. It’s not that they changed for me, but I changed because I came to Japan. I will change by accepting the Japanese way*.” (EPA3)

Even when they had language problems or work-related frustrations, they found ways to relieve stress, such as exercising or cleaning, and tried to persevere. Even when the location of their assignment was not in an urban setting and was sometimes inconvenient, they gradually became familiar with it and developed an attachment to it because they had chosen an assignment in an environment similar to that of their hometown. In many cases, they liked the Japanese culture, were interested in it, and had amicable feelings towards Japan.

However, there was confusion about participants who did not have previous nursing experience in their home countries, the differences in nursing practices in Japan compared to their home countries, and the unique Japanese culture. They blamed themselves for not wanting to cause trouble for their fellow nurses and not noticing patients’ distress. In addition, there were cases where the nurses felt that they had no choice but to solve their emotional problems, even when they were in pain and could not talk to anyone about their problems.

“*If I have any problems, I will ask my preceptor on days when my preceptor is there, though. But my preceptor also has her own patients to take care of, so I will be a bother to her. I would bother them. I did not like that*.” (EPA 3)

### Support from EPA peers and family

In some cases, the participants decided to come to Japan at the invitation of a senior colleague or with a friend. After coming to Japan, they bonded with people from their home country who worked at the same hospital and supported each other (i.e., referring to interactions with fellow EPA nurses and caregivers). However, it was also true that they spoke in their native language with people from the same country, which reduced their opportunities to communicate in Japanese.

Although the participants experienced a solitary life and the hardships of studying in Japan, they were in frequent contact with their families living in their home countries via video calling and other means and had the understanding and support of their families. Participants living with a partner from the same country had more personal time. The presence of a partner was important for emotional support. Before the COVID-19 pandemic, temporary visits to their home countries were a source of energy for continuing to work in Japan. In some cases where siblings worked in other parts of Japan, they supported each other.

*“My preceptor is X senior, who is from the same country. It was great, I was happy. I was really happy. Even if I do not understand something, it is difficult to express what I want to say to other Japanese nurses*.” (EPA5)

However, some participants felt lonely because their trusted EPA colleagues had returned to their home countries or moved to other regions or because they were separated from their children in their home countries. In some cases, they experienced considerable difficulty balancing child rearing and work in Japan without family support in their home country.

When family members opposed their coming to Japan, they eagerly hoped the participants would return home anytime. They had seen several seniors return to their home countries due to certain family circumstances there. In the case of single participants, they were not planning to marry a Japanese man but wanted to marry a man from their country, and it was unclear whether they would actively continue to reside in Japan. In most cases, they had no family in Japan and had little contact with Japanese people; as such, they were fighting loneliness every day.

“*In our ward, there was a Filipino nurse named Ms. Y, but she was gone when I came. I am the only Filipino working in the ward, and I feel lonely*.” (EPA1)

### Acceptance by others

For EPA nurses, acceptance by Japanese patients and colleagues was a great motivating factor; they worked hard despite language difficulties and gradually became accepted by the patients. They were truly happy to be trusted by the patients, as they were entrusted to care for them in the same way or individually as the Japanese nurses. In particular, the presence of trusted Japanese people, such as the nursing director and colleagues, encouraged them to continue working at the facility and significantly impacted their work and daily lives. Some EPA nurses interacted with them outside of work and were truly grateful for their warm and supportive presence as if they were family.

“*What makes me happiest is when patients leave the hospital, they say, ‘Z-san (EPA nurse’s name), you are so kind, thank you, thank you for always taking care of me’. The patient acknowledged me, and that made me happy. The patient did not remember the name of the Japanese nurses, but she remembered my name, and that made me happy.*” (EPA6)

However, it was also true that when they were new to Japanese nursing practice, patients often rejected them, especially because they were foreign nurses, which made them fear their interactions with patients and caused them emotional distress. Furthermore, only a few of the EPA nurses had close Japanese friends, and it was also true that they found it difficult to build relationships with Japanese people.

“*Sometimes, patients told me that I was a foreigner and could not communicate, so they said, ‘You are not good enough, please call a Japanese nurse’. Such instances hurt my heart a little*.” (EPA6)

### Satisfaction with the support received

The EPA nurses were attracted by the availability of study time when selecting a facility, and the availability of ongoing study support was important to them. It was important to have ongoing Japanese language study support, not only until passing the national exam but also after passing the exam. Further, they were grateful for facilities that provided support for daily life concerns, including administrative procedures for residency and family-related matters.

*“First, this hospital provided great support for my studies, and thanks to that,I was able to pass the national nursing exam. Even after passing the exam, there is support and ongoing study support. The staff on the wards are also very attentive and teach me how to do my job*.” (EPA2)

However, in some cases, EPA nurses were considering moving to another hospital or area where they could earn a better income than they currently do to sustain life in Japan with their future partners. These women were unsatisfied with their income in Japan but felt better off than nurses in their home countries regarding income and work arrangements. Some EPA nurses were anxious that their generous study support would suddenly disappear until they passed the national exam and that they would be expected to stand independently on the job as other Japanese nurses did.

When they first arrive in Japan, EPA nurses often cannot speak, read, or write in Japanese. In addition, they come from tropical regions, and they are sometimes affected by the cold winters in Japan and the lack of adequate transportation services in some cases, which can hinder their daily lives. Furthermore, to work-related considerations, the nurses stressed the need for warm and attentive lifestyle support to help foreign nurses adjust to their lives in Japan.

“*The Nursing Director has been a great help to me in so many ways. I can talk to her about anything, like going shopping. So, it is a great help. My friends in other hospitals have to study in their own time. Here, I work half a day and study half a day. I thought it was great*.” (EPA2)

“*At first, emotional support is important; the EPA nurses are all from different countries, so we are feeling sadness, the culture is different, and the food may not be right. In the beginning, we may not be able to eat until we get used to it. That is why we want you to create a warm environment for us to get used to. I want them to understand like that.*” (EPA4)

### Motivation

The EPA nurses participated in the program because they wanted to achieve their dreams and were proud of the considerable effort they had put in to pass the national exam. They were eager to do their best and learn about Japan’s high level of medical care because they decided to become a nurse in Japan, even if it was difficult.

They were interested in how their Japanese colleagues treated patients and were willing to learn how to care for them. By working diligently, they gradually began to be entrusted with nursing tasks and could gradually stand independently while working. The joy of being trusted motivated them. They had a strong desire not to cause trouble for the staff around them, and through hard work, they gradually became able to handle their duties independently. They sometimes thought about advancing their careers in Japan. An eventual sense of familiarity with work and life in Japan led to the desire to continue working in Japan. Some participants said they could work hard because they aimed to educate their children in Japan.

“*I want to work in Japan for a long period of time, so right now, the most important thing is to take good care of my patients and to be able to apply my nursing skills as well as possible.*” (EPA5)

## Discussion

**Fig. 1:**
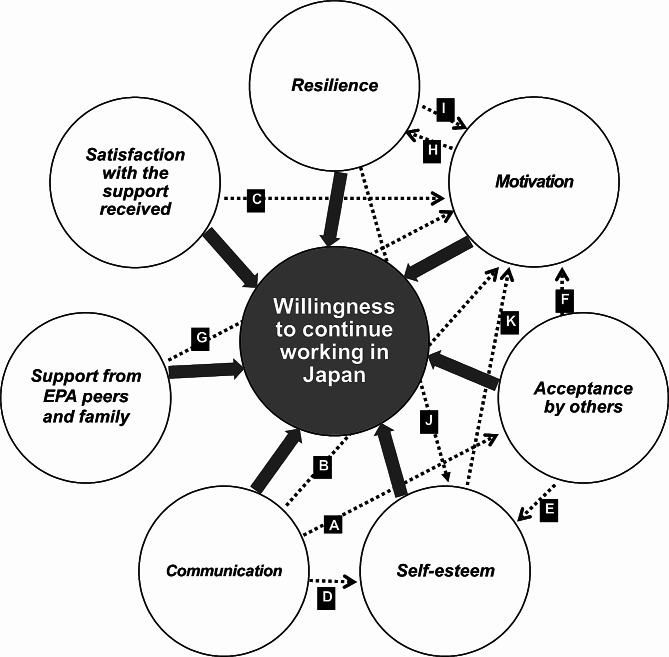
Thematic map of factors associated with EPA nurses’ willingness to continue working in Japan. *Note* Seven themes emerged from the thematic analysis. These themes, shown in italics, include: (1) communication, (2) self-esteem, (3) motivation, (4) resilience, (5) support from EPA peers and family, (6) acceptance by others, and (7) satisfaction with support received. Bold arrows indicate the direction of the factors associated with willingness to continue working in Japan. Dotted arrows indicate the potential direction of influence between the themes

This qualitative study used an interview survey design to identify seven themes. The following seven themes were identified as factors associated with the willingness of EPA nurses to continue working (Fig. 1: bold arrows), and it was suggested that there existed a connection between these themes (Fig. 1). The theme of ‘communication’ was linked to ‘acceptance by others’ and ‘self-esteem’ (Fig. 1: A, D). Additionally, ‘acceptance by others’ was associated with ‘self-esteem’ (Fig. 1: E). ‘Resilience’ was found to be connected to ‘self-esteem’ (Fig. 1: J). Furthermore, ‘motivation’ was linked to ‘resilience’ (Fig. 1: H). The themes of ‘communication’, ‘self-esteem’, ‘resilience’, ‘support from family and peers’, ‘acceptance by others’, and ‘satisfaction with support received’ were all linked to ‘motivation’ (Fig. 1: B, C, F, G, I, K).

### Need for continuous learning support

The study participants were Filipino and Vietnamese, and their periods of stay in Japan varied. Of the participants, eight passed the Japanese national nursing exam. The passing rate of EPA nurse candidates in Japan for the 2023 national exam was 22.4%, the highest ever. The passing rate for Vietnamese candidates was 45.7%, Filipino candidates was 15.9%, and Indonesian candidates was 11.5%, with the high passing rate of Vietnamese candidates standing out [25]. The number of Vietnamese nurses is also expected to increase soon. Although the participants have passed the national exam, their Japanese language proficiency is not uniform. All the participants received Japanese language learning support from their respective hospitals and study support for the national exam until they passed it. However, after passing the national exam, they were considered to be just another nurse among the staff, so they went about their daily work with a sense of not wanting to cause trouble to other staff and with anxiety about having to stand on their own, and this stress was evident. In particular, it was difficult for some participants to express their opinions at conferences, develop nursing plans, and write nursing records in Japanese. All the participants experienced difficulty communicating with patients, family members, and colleagues in daily work. Participants felt unaccepted by patients and colleagues due to communication problems (Fig. 1: A), which further reduced their motivation (Fig. 1: B). On the other hand, participants were motivated by the perceived improvement in their ability to communicate in Japanese with patients and colleagues (Fig. 1: B). A prior study of Indonesian EPA nurses has also reported communication challenges with patients and colleagues. In this study, Vietnamese and Filipino EPA nurses faced similar challenges. However, the participants in this study received Japanese language learning support until they passed the national nursing exam. The study found that they were worried that the language support would stop after they passed the exam. Before coming to Japan, the participants selected facilities based on the availability of study support for Japanese language learning and national exams rather than high salaries; study support was an important factor. However, study support after passing the national exam varies across facilities. When this support is lost, the nurses must become independent, and some participants were very anxious about the loss of learning support. (Fig. 1: C). Communication with patients, colleagues, and other professionals is of utmost importance for nurses whose job is to provide interpersonal assistance; however, in cases where no time had passed since passing the national exam, this was a challenge. All of these difficulties create emotional stress and frustration, leading to feelings of isolation, loneliness, low-self-esteem (Fig. 1:D); indeed, Magnusdottir highlights nurses’ communication barriers and experiences of discrimination and alienation as personal challenges [26].

### Tolerance for a diverse workforce

In this study, in some cases where the study participants had only Japanese nurses as colleagues, they felt that patients and colleagues did not accept them because they were not Japanese, which deepened their sense of isolation and loneliness as a minority group member. This led to decreased self-esteem and motivation (Fig. 1: E, F), and a, desired for workplace with a diverse workforce. In some cases, they considered moving to a country where English is spoken or to an area in Japan with many foreign nurses. This suggests that the movement of EPA nursing personnel to other countries is inevitable if they can more easily demonstrate their abilities to work there than in Japan. For those who were not accustomed to life in Japan after arriving, building relationships with Japanese colleagues was difficult, and they had to endure loneliness and anxiety daily. Although there was encouragement from family members in their home countries and from fellow EPA members, only two participants were living with their families. This study found that having the same EPA peers was important. In some cases, the presence of seniors from the same country was the reason for coming to Japan, and they relied on seniors who were active in Japan as role models. They were also motivated by the encouragement they received from their EPA peers (Fig. 1: G).

According to prior research, some nurses who moved abroad reported feelings of isolation, loneliness, coping difficulties, frustration, confusion, and loss of confidence and self-esteem during the adjustment process [27]. A step to mitigate these issues involves welcoming diversity in the nursing workforce, which could lead to better workplace conditions.

Globally, foreign-educated nurses are reported to be important contributors to the workforce, bringing important skills to healthcare systems. In Japan, a project to accept foreign nurses, based on an EPA, began in 2008 [28]. However, as of 2023, only 648 nurses have passed the national nursing exam under this program [8]. Although Japan’s national exam passing rate is increasing, it is still not high, limiting its acceptance of foreign nurses compared to Western countries. A study of Japanese hospital managers who had never hired EPA nurses found that 90% of hospital managers thought it would be difficult to train EPA nurses, and half of them were interested in Japan’s EPA nurse recruitment policy, but only 20% were willing to hire EPA nurses in the future [5].

The international migration of nurses is increasing in magnitude. Globally, in 2018−2019, approximately 3.7 million nurses (one in eight nurses) worked outside their country of birth, mostly in high-income countries. The number of foreign nurses working in OECD countries increased by 20% in the five years from 2011 to 2016 [29, 30].

As the number of foreign residents in Japan increases, healthcare needs are expected to diversify. Regarding residence status, permanent residents accounted for 28.1%, followed by technical interns at 10.6%, the second highest percentage, with the largest number of technical trainees coming from Vietnam [2]. It has been reported that internationally educated nurses are a valuable resource and enrich the diversity of healthcare teams [31]. Effective use of EPA nurses may be beneficial in meeting the diverse healthcare needs of foreign residents, including technical interns, and will lead to efforts to create opportunities and a climate of mutual recognition and acceptance of diverse human resources.

In addition, the presence of nursing managers, such as directors of nursing and chief nurses, who are dedicated to fostering a diverse nursing workforce, was considered to have a significant impact on EPA nurses’ decision to continue working. This is especially true as EPA nurses had difficulty building relationships with Japanese people around them. The managers took the initiative to warmly support the EPA nurses as if they were family, not only in their work but also in their daily lives. This support was considered as a major factor in motivating EPA nurses to continue working in Japan.

Foreign-educated nurses have been reported to value recognition and appreciation from patients, colleagues, and employers [17]. Similarly in this study, EPA nurses felt pleasure in receiving recognition from patients and colleagues, which was thought to boost their self-esteem, increase their job satisfaction, and improve their motivation (Fig. 1: E, F).

### Increasing resilience

In this study, the participants were highly motivated and flexible to learn Japanese nursing practices, even though that the techniques and tasks differed from those in their respective home countries (Fig. 1: H). They were a highly educated group, had clinical experience in their home country, and had good self-esteem as nurses. It was thought that individuals who were able to adapt to the challenges of loneliness and embrace the differences of their home country were more likely to maintain their motivation to work in Japan (Fig. 1: I). However, other staff’s harsh verbal instructions and attitudes damaged EPA nurses’ self-esteem (Fig. 1: D) and lowered their motivation (Fig. 1: B). Previous research has reported that feeling disrespected and unappreciated of nurses’ past experiences can lead to frustration and demoralisation [32–34]. Filipino nurses who moved abroad reported feeling like outsiders, as their previous clinical experiences were not respected, and they were often put in a position to receive guidance from local nurses [35].

Some EPA nurses used their stress-busting techniques to cope with these challenging situations and stay motivated (Fig. 1: I); however, if these are not overcome, they can lead to low self-esteem (Fig. 1: J), which can, in turn, lead to low motivation (Fig. 1: K) and mental health problems. The resilience of EPA nurses appeared to influence whether they would continue to work in Japan, but the level of resilience varied among individuals. According to a previous study, individual protective factors associated with resilience include hopes and dreams for a better economic and social life and a future for oneself and one’s family. Personal strengths include learning, networking, finding support, praying, relying on inner confidence, gaining social recognition and respect, and ignoring discrimination. Emphasising courage and pride as personal protective factors, the authors emphasise that nurses educated in foreign countries take pride in their knowledge, experience, abilities, work skills, and sense of being needed in the workplace [36].

In this study, we did not extract the thematic names of the cultural differences that were reported in a previous study of Indonesian EPA nurses in Japan [7]. However, we extract a new theme of ‘satisfaction with the support received’ to overcome cultural barriers such as language, climate, and lifestyle, which we considered a crucial factor in their decision to work in Japan. The participants were flexible in accepting Japanese nursing practices, eager to learn Japan’s finely tuned nursing qualities, and committed to career advancement. The findings suggest that EPA nurses need to be recognised and motivated by their peers and managers.

### Limitations

Our study, which employed a qualitative approach, uncovered various themes pertaining to EPA nurses’ decision to continue working in Japan. However, it is important to note that our analysis is based on a limited amount of data. Our findings indicate that there are several intricate factors that impact their willingness to stay, and additional quantitative research should be conducted to delve deeper into these factors. This will allow for a better understanding of the complexities surrounding their decision to continue working in Japan.

In this study, EPA nurses currently working in Japan were included. Based on their experiences, their perspectives may help clarify the support foreign-educated nurses need. However, this study did not include host-country stakeholders in the study sample. Further research is needed from both perspectives, including host-country stakeholders receiving nursing staff.

## Conclusion

After arriving in Japan, the EPA nurses studied hard, intending to pass the national exam and obtain their nursing licenses. However, even after becoming nurses, they experienced many difficulties and tended to isolate themselves. This study found that they needed not only support from their colleagues and superiors but also an understanding of EPA nurses from Japanese society. In today’s globalised world, Japanese nursing practices must involve an understanding of the diversity of the nursing workforce; to make the most of the valuable nursing talent of EPA nurses, it is necessary to build a support system so that they can continue to have pride in and motivation towards their work.

Although the rate of EPA nurses passing the national exam is increasing, some EPA nurses return to their home countries, and the retention rate of EPA nurses in Japan is poor. However, few studies have been conducted on EPA nurses in Japan. The international mobility of nurses is expected to expand further, and the demand for foreign nurses is expected to increase in many countries. Further research is needed to ensure that foreign nurses receive a fair evaluation in the host country and to encourage them to continue working there.

### Electronic supplementary material

Below is the link to the electronic supplementary material.

Supplementary Material 1

## Data Availability

The datasets used and/or analysed during the current study are available from the corresponding author on reasonable request.

## Abbreviations

EPAs: Economic Partnership Agreements
COREQ: Consolidated criteria for reporting qualitative research
OECD: Organisation for Economic Co-operation and Development
