# Supplementary material for: Factors associated with foreign-educated nurses’ willingness to continue working in Japan: a qualitative study
Source: BMC Nurs. 2024 Apr 3;23:228. doi: 10.1186/s12912-024-01890-4 (PMC10988908; doi:10.1186/s12912-024-01890-4)
Supplement: Supplementary file 1 — Supplementary Material 1 [file 12912_2024_1890_MOESM1_ESM.docx]

**Interview guide**

We will ask about the following points, while making sure that the participant is able to speak

as freely and spontaneously as possible.

**Warm up**

1. Could you describe why you joined the EPA Nursing Acceptance Project?

2. Why did you choose this hospital?

**Questions**

1. Please tell me what type of nursing practice you are currently involved in?

- Do you have any feelings of confusion about the differences in nursing practice between Japan and your country?

1. Please tell me what confusion or difficulties you have encountered in carrying out your nursing duties in Japan.

- How did you overcome them?

3. Please tell me who you consult when you have difficulties in your work in Japan.

- Can you ask your colleagues for help when you have a problem with a patient or their family?

4. What was the most impressive or enjoyable part of your nursing experience in Japan?

5. Do you still have problems with language when working as a nurse in Japan? If so, please tell me when.

6. Please tell me how you cope with cultural adjustment when working in Japan?

7. Could you describe how you take care of your mental health to work as a nurse in Japan?

8. What do you expect from your colleagues, superiors and us Japanese to continue working in Japan in the future?

9. Do you want to continue working as a nurse in Japan? Please tell me why you think so.
